# Benefits of maternal pectin supplementation in gestation diet on vaginal microbiota of sows and intestinal health of newborn piglets

**DOI:** 10.3389/fvets.2024.1392399

**Published:** 2024-06-04

**Authors:** Jiaqi He, Jie Zheng, Yingyan Huang, Shuang Li, Lun Hua, Xuemei Jiang, Lianqiang Che, Zhengfeng Fang, Bin Feng, Yan Lin, Shengyu Xu, Jian Li, De Wu

**Affiliations:** Key Laboratory for Animal Disease-Resistant Nutrition of the Ministry of Education of China, Institute of Animal Nutrition, Sichuan Agricultural University, Chengdu, China

**Keywords:** intestinal health, newborn piglet, pectin, sow, vaginal microbiota

## Abstract

Pectin is a proven prebiotic and widely used in human health products. This study aims to assess the impact of dietary pectin supplementation during gestation on sow vaginal microbiota and the offspring's intestinal composition. Thirty sows were randomly allocated to two groups and fed a standard diet (CON) or a standard diet supplemented with 3 g/kg pectin (PEC). Blood, feces, and vaginal swab samples from the sows and blood, intestines issue, and colonic content samples from the offspring were collected and analyzed. The results indicate that the relative abundance of vaginal *Lactobacillus* was notably enhanced in the PEC group and fecal β-glucuronidase (β-G) activity and plasma 17β-estradiol (E2) concentration were also significantly increased in the PEC group. Newborn piglets were found to host different microbial communities as well. At the phylum level, Proteobacteria dominated in the CON group, and Firmicutes was predominant in the PEC group. Newborn piglets in the PEC group had a lower interleukin-6 (IL-6) concentration in their plasma. The expression of intestinal cytokines of offspring was improved as well. Villus height and villus height/crypt depth (V/C) in the PEC group were extremely higher than those in the CON group. In conclusion, dietary pectin supplementation can be of benefit to both sows and newborn piglets.

## 1 Introduction

It has been reported that the vaginal microbiota plays an important role in the health of the maternal reproductive tract (1). Most of the times, vaginal bacteria were found to cause reproductive tract infections like endometritis or bacterial vaginitis (2). These bacteria can harm sows and newborn piglets as well (3), leading to serious economic losses. Our group also found that vaginal microbiota were associated with the performance of the sows and their inflammatory cytokines (4). Although only a few studies are available that characterize the vaginal microbiota and their development in pregnant sows (5), studies in humans have revealed that vaginal *Lactobacillus* have the ability to resist pathogenic bacteria and maintain the vaginal microecological balance (6–8). Moreover, the relative abundance of vaginal *Lactobacillus* is altered by blood estradiol concentration (6, 7, 9). Beta-glucuronidase (β-G) secreted in the gut can deconjugate the conjugated estrogens into aglycone estrone and 17β-estradiol (E2) (10). Diets richer in dietary fiber were associated with lower odds of bacterial vaginitis (11) and fermentable dietary fiber can increase cecal β-G activity as well (12). Pectin, a fermentable dietary fiber extracted from fruits (13), is considered an ideal dietary fiber for both humans and animals. Studies in weaned pigs, rats, and humans *in vitro* or *in vivo* have shown that dietary pectin supplementation can alter intestinal and fecal microbiota as well as improve systemic anti-inflammation (13–16). Moreover, studies suggested that supplying pectin to rats can increase the activity of cecal β-G and pectin is confirmed to contribute to an increase of β-G activity as well (15, 17–19).

Therefore, the objectives of this study were to investigate the effects of dietary pectin on vaginal microbiota of sows in their final stage of gestation. At the same time, we were also interested in knowing if the gestational dietary supplementation of sows with pectin would benefit the newborn piglets.

## 2 Materials and methods

### 2.1 Ethical approval

This experiment was conducted at the Research Farm of Animal Nutrition Institute, Sichuan Agricultural University, Ya'an, China. The experimental protocol used in the present study followed the current laws regarding animal protection (Approval No. 20200156).

### 2.2 Animals and diets

This study utilized 30 large white × landrace crossbred sows, which were closely matched in terms of body weight (228.16 ± 6.16 kg), backfat thickness (12.88 ± 0.26 mm), and parity (5–7). Following artificial insemination, sows were randomly divided into two groups: control (CON) or pectin (PEC). Each group contained 15 replicated pens, each of which accommodated one sow. The CON diet was formulated on a corn and soybean meal diet to provide 3.38 Mcal digestible energy/kg and 12.08 g/kg total dietary fiber according to the nutrient requirements of gestating sows of National Research Council (NRC) (20) (Table 1).

**Table 1:** Composition of gestation diet and nutrient level (air-dry basis) %.

**Ingredients**	**Content, %**
Corn	79.59
Soybean meal	14.0
Fishmeal (CP67%)	1.50
Soybean oil	1.50
CaHPO_4_	1.20
Limestone	1.10
NaCl	0.40
L-Lys HCl	0.08
L-Threonine	0.08
Choline chloride	0.15
Vitamin and minerals premix^a^	0.40
Total	100.0
**Nutrient composition**	
DE, Mcal/kg	3.38
NE, Mcal/kg	2.52
CP, %	14.05
EE, %	4.59
CF, %	1.74
Ca, %	0.88
Available P, %	0.38
SID- Lys, %	0.65
SID-Met, %	0.20
SID-Thr, %	0.51
SID-Trp, %	0.13

^a^Mineral premix provided per kilogram of diet: Cu (CuSO_4_·5H_2_O) 6 mg; Fe (FeSO_4_·7H_2_O) 100 mg; I (KI) 0.14 mg; Zn (ZnSO_4_·7H_2_O) 100 mg; Mn (MnSO_4_·H_2_O) 20 mg; Se (Na_2_SeO_3_·5H_2_O) 0.3 mg. Vitamin premixes provided per kilogram of diet: vitamin A, 3,000 IU; vitamin D3, 1,000 IU; vitamin E, 8 IU; vitamin K3, 1 mg; vitamin B1, 1 mg; vitamin B2, 2.5 mg; vitamin B6, 1.2 mg; vitamin B12, 0.12 mg; nicotinic acid, 10 mg; pantothenic acid, 5 mg; folic acid, 0.5 mg; biotin, 0.5 mg.

The gestational diets were meticulously crafted to maintain consistent nutrient content across all groups. For the CON group, the feeding regimen varied by gestation stage: 2.4 kg/day from 0 to 30 d, 2.3 kg/day from 30 to 90 d, and 2.7 kg/day from 90 to 110 d of gestation. In contrast, sows in the PEC group were fed a diet supplemented with 30 g/kg pectin. The scale of feeding in this group was 2.47, 2.37, and 2.78 kg/d at different stages of gestation. Sows were fed at 08:30 and 14:30 h, and water was provided *ad libitum*. On day 110 of pregnancy, all sows were cleaned and transferred to individual farrowing pens equipped with slatted floors and warm pads for the newborn piglets. To standardize litter sizes to 8–12 piglets per nest, cross-fostering was performed within 24 h post-farrowing, exclusively among sows from the same treatment group. Lactation feed intake began at 2 kg/day, gradually increasing by 0.5 kg/day for the first 5 days post-partum, thereafter allowing *ad libitum* diet access. Both sows and piglets had unrestricted access to water. All piglets were weaned on day 21 of lactation.

### 2.3 Sample collection

#### 2.3.1 Growth performance of suckling piglets

The weight of the piglets was recorded individually at 10:00 h on 7, 14, and 21 d of lactation.

#### 2.3.2 Vaginal samples

To avoid contamination of the vaginal samples, a specially designed double-guarded swab equipment was used. In brief, a sterile swab was placed inside a sterile plastic tube, the front of which was covered with a sterile plastic wrap to protect the swab. A sterile thin stick, inserted from the rear, was used to push the swab forward. The outer area of perineum was disinfected with 75% ethanol. Then a vaginal dilator, which was used to open the vulva, facilitated the swab's insertion into the vagina. To prevent contamination of the swab from the front vaginal area, the pipette, still covered by the plastic sheet, was guided deeper into the deep vagina. Once in the deeper vagina, the plastic sheath was broken by pushing the thin stick, exposing the swab. The swab remained in the deeper vagina for 1 min to ensure a thorough exposure to vaginal secretions. Afterward, the swab was carefully withdrawn from the tube and removed, while the tube itself was kept inside the vagina to prevent contamination. The swabs were then placed in sterile microtubes and stored at −80°C until further processing. This entire procedure was conducted on day 110 of gestation, following the transfer of the sows to the farrowing crates.

#### 2.3.3 Blood samples

Fasted blood samples were collected from ear veins from each sow before the morning meal on day 110 of gestation. Blood samples (10 mL) were collected into heparinized tubes, kept at 4°C for 30 min, and then centrifuged for 15 min at 3,000 × *g* at 4°C. Plasma samples were harvested and stored at −20°C until analysis.

#### 2.3.4 Newborn piglet samples

At birth, a total of 12 newborn male piglets (*n* = 6 per group) from different litters, without consuming colostrum, with body weight close to the average in each treatment were slaughtered for sampling. Approximately 10 mL of blood was drawn from each pig via jugular vein venipuncture. Blood samples were kept at 4°C for 30 min, centrifuged at 3,000 × *g* for 15 min at 4°C as mentioned above. The contents of the colon were collected in sterile tubes and stored at −80°C for 16S rRNA analysis. The jejunum, ileum, cecum, and colonic tissues of the piglets were separated on a sterile workbench. The middle portions of these tissues were then sectioned, immediately frozen in liquid nitrogen, and stored at −80°C for quantitative polymerase chain reaction (qPCR) test. Meanwhile, the intestinal segments (duodenum, jejunum, and ileum) were fixed in 4% paraformaldehyde for at least 24 h, dehydrated, cleared, and paraffin embedding were performed. Then serial sections of 5 μm thickness were made, and this was followed by hematoxylin and eosin staining. Two transverse sections of each intestinal sample (duodenum, jejunum, or ileum) were prepared on one slide for morphometric analysis. A total of 12–20 intact, well-oriented crypt-villus units per sample were chosen randomly and measured. Villus height measurements were taken from the tip to the base of the villus between individual villus, and crypt depth was measured from the valley between individual villi to the basal membrane. The small intestinal crypt depth (μm) and villus height (μm) were measured with JD801 morphologic image analysis software (JEDA Science-Technology Development Co., Ltd., Nanjing, China), and then villus height/crypt depth (V/C) was calculated as the villus height divided by the crypt depth.

### 2.4 Analysis of fecal β-G

A commercial ELISA kit (Meimian, Jiangsu, China) was used for determining the fecal β-G activity. The feces samples were pretreated to meet the requirements of the kit. Firstly, 9 mL of phosphate-buffered saline (PBS) was added to 1 g of feces and homogenized by vortex. Then, mixtures were centrifuged at 2,500 × *g* at 4°C for 20 min to collect the supernatants. Finally, supernatants were measured for β-G activity with a kit.

### 2.5 Analysis of plasma 17β-estradiol

A commercial ELISA kit (R&D Company, USA) was selected to measure the concentration of 17β-estradiol in the plasma of sows on day 110 of gestation.

### 2.6 Vaginal *Lactobacillus* flat colony culturing and counting

Within a sterile laminar flow cabinet, swabs previously stored at −80°C were removed and immersed in 10 mL precooled PBS within 15 mL centrifuge tubes for 30 min to ensure the complete elution of the vaginal secretion on swabs. The resultant liquid was then diluted at 10^−7^ in PBS. From this dilution, 10 μL was decanted into 15 mL Man Rogosa Sharp (MRS) agar (Hopebiol, Qingdao, China) and subsequently incubated anaerobically at 37°C for 72 h. Anaerobic conditions were preserved using anaerobic atmosphere generation bags (Hopebiol, Qingdao, China) in Anaero Jars (Fisher, Hampton, NH, USA). After 72 h of incubation, the resulting colonies were enumerated, and the counts were expressed as the decimal logarithms of the colony-forming units per milliliter (log CFU/mL).

### 2.7 Microbial analysis

The microbial diversity was determined as described in an earlier research from our group (21). In brief, the bacterial genomic DNA from frozen sow stool, vaginal swabs, and colon contents of newborn piglet were extracted using the DNA kit (Omega Bio-Tek, Norcross, GA, USA). Subsequently, Majorbio Company (Shanghai, China) performed the sequencing and data analysis on the Illumina HiSeq 2000 platform. The same operational taxonomic unit (OTU) was defined as sequence similarity reaching 97%. The taxonomy of each OTU was conducted with Ribosomal Database Project (RDP) classifier program against the Silva 16S rRNA database (Release138: http://www.arb-silva.de). Categorical data was compared by calculating α-diversity and β-diversity. The Simpson and Shannon indexes were used to determine the difference of α-diversity. The principal coordinate analysis (PCoA) based on weighted Unifrac distance matrix was used to summarize the β-diversity (22, 23) Analysis of similarities (*Anosim*) was used to access differences among the microbial communities. All analyses from clustering to α- and β-diversity were performed in QIIME (V1.9.1) and displayed in R software (V3.3.1).

All obtained raw sequence datasets have been uploaded to the NCBI Sequence Read Archive (SRA) with the accession number PRJNA754492.

### 2.8 Analysis of plasma inflammatory cytokine

Commercial ELISA kits (MEIMIAN, Jiangsu, China) were used for determining the concentrations of plasma IL-1β, IL-6, IL-10, and TNF-α of newborn piglets following respective instructions.

### 2.9 Measurement of gene expression

A quantitative real-time polymerase chain reaction (qPCR) was used to analyze mRNA expression levels of IL-1β, IL-6, IL-8, IL-10, and TNF-α in piglet intestinal samples. The specific primer sequences used for the real-time PCR analysis are listed in Supplementary Table 1. β-Actin was utilized as an internal control, co-amplified alongside the target genes to facilitate normalization and quantification of gene expression. All samples were measured in duplicate. The relative mRNA abundance of the genes detected in the intestinal samples was calculated with the 2–^ΔΔCt^ method.

### 2.10 Statistical analysis

The data shown in the study were expressed as mean ± standard error of the mean (SEM) or mean with cluster standard errors. The activity of sows fecal β-G, sow plasma E2, and gene expression of cytokines in jejunum, ileum, cecum, and colon of piglets were analyzed by an independent-samples *T*-test after normality tests. Statistical significance was assessed by SPSS 22.0 (IBM SPSS Company, Armonk, NY, USA), and a value of *P* < 0.05 was considered statistically significant, whereas 0.05 ≤ *P* < 0.10 was considered a tendency.

Normality tests were performed on the microbial data at first. If the data fit the normal distribution, Student's *t*-test will be used for analysis. If not, statistical analyses for the relative abundance of the vaginal and fecal microbiota will be performed with Wilcoxon rank-sum test with R (Version 3.3.1) stats package and Python scipy package. Statistical significance was considered for a value of *P* < 0.05, whereas 0.05 ≤ *P* < 0.10 was considered a tendency.

## 3 Results

### 3.1 Concentration of E2 in sow plasma

**Figure 1:**
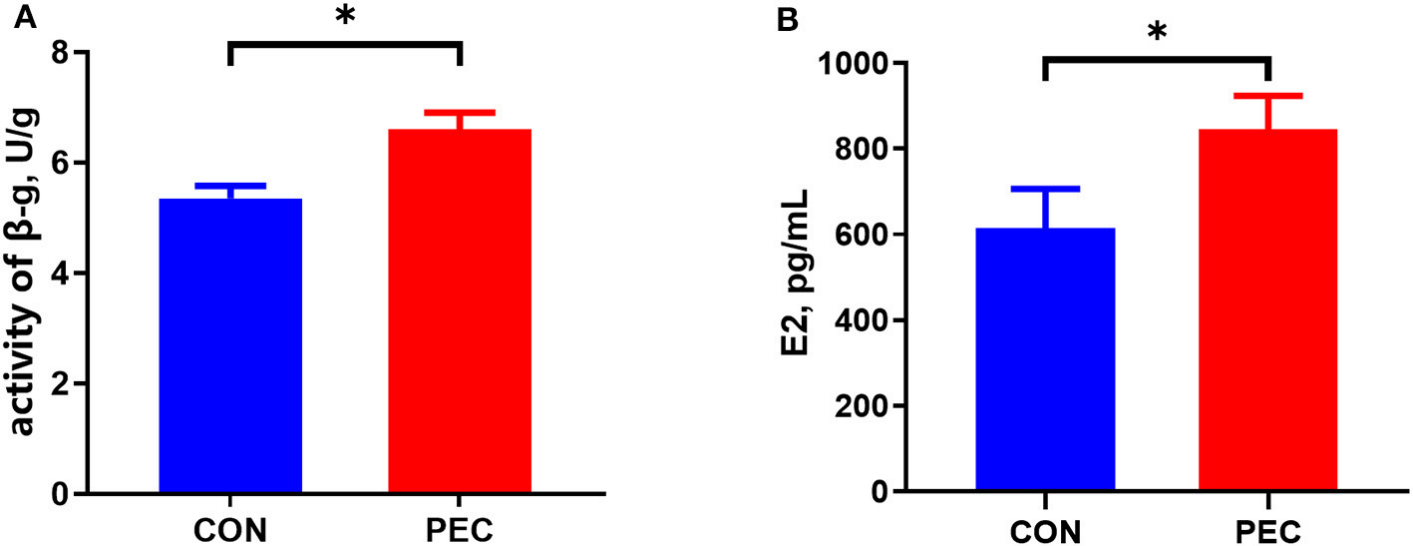
Effects of dietary pectin in pregnant sow diets on the plasma E2 **(A)** and fecal β-G **(B)**. CON, control group; PEC, pectin group. *n* = 15. ^*^P < 0.05.

The concentration of E2 in plasma of sows in the pectin group was substantially higher than that in the control group at day 110 of gestation (*P* < 0.05, Figure 1A).

### 3.2 Activity of β-Glucuronidase in sow feces

Dietary supplementation with pectin for sows during pregnancy significantly increased the activity of β-G in feces (*P* < 0.05, Figure 1B).

### 3.3 Changes in sow vaginal microbiota

**Figure 2:**
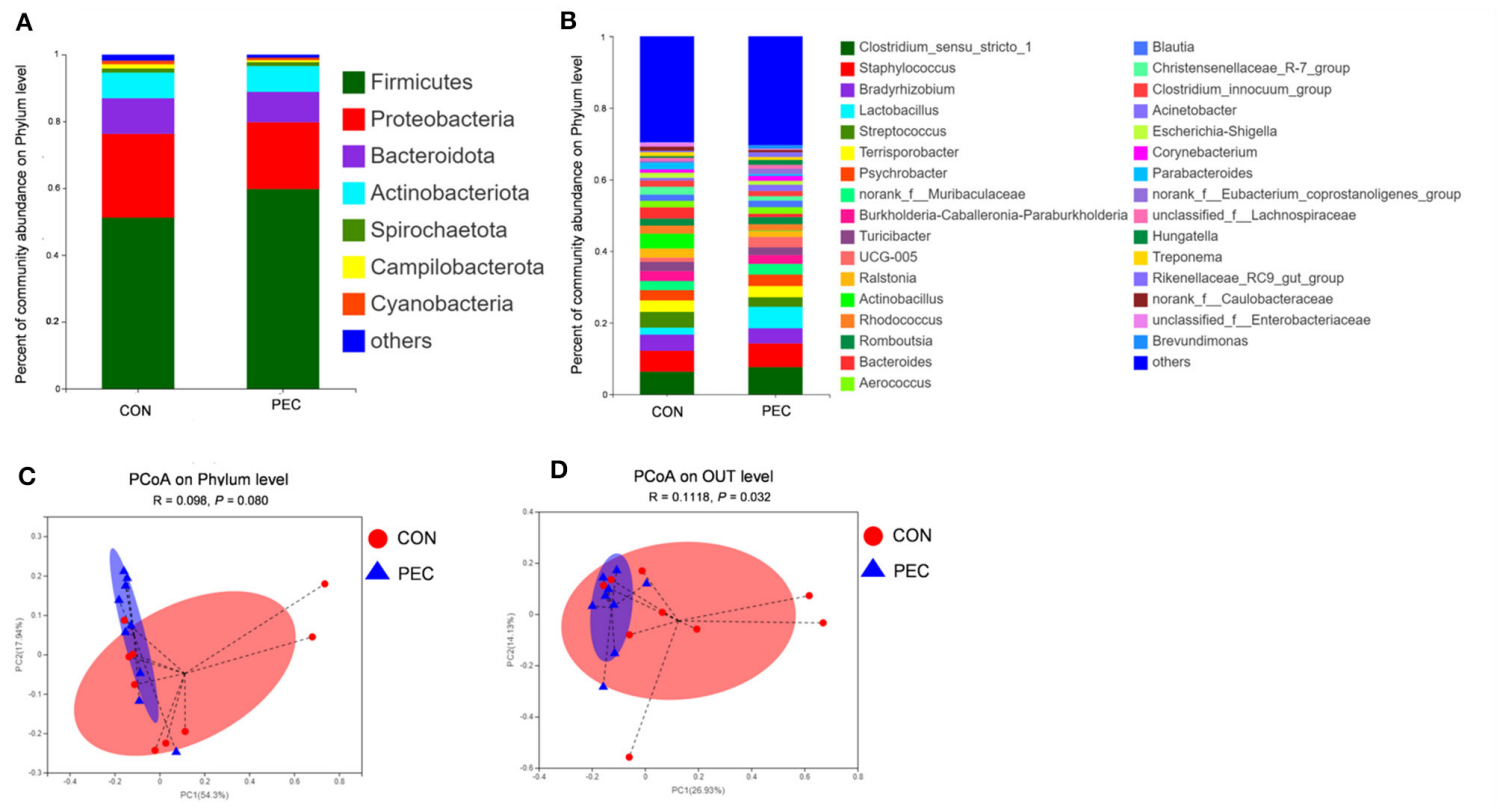
Comparison of the composition of sow vaginal microbiota on day 110 of gestation. **(A)** Plot diagram of the relative abundance of microbiota at the phylum and genus levels **(B)**. Phylum level **(C)** and OTU level **(D)** PCoA profile based on the weighted Unifrac distance of the OTUs in each sample on day 110 of pregnancy. CON, control group; PEC, pectin group. *n* = 9.

The top *four* dominated phyla were Firmicutes, Proteobacteria, Bacteroidota, and Actinobacteriota in both the groups (Figure 2A). Bar plots (Figure 2B) were created to illustrate the ratio of sow vaginal microbiota at genus level at day 110 of gestation. Genus *Lactobacillus* was present to the extent of 5.72% in the PEC group but only 1.85% in the CON group.

PCoA was used to analyze the β-diversity between samples. No difference was observed at the phylum level, while significant differences were found at both species level and OTU level (Figures 2C, D). At 110 days of gestation, the vaginal microbiota of PEC sows were closer within the group, while the distribution was more discrete in the CON group.

**Figure 3:**
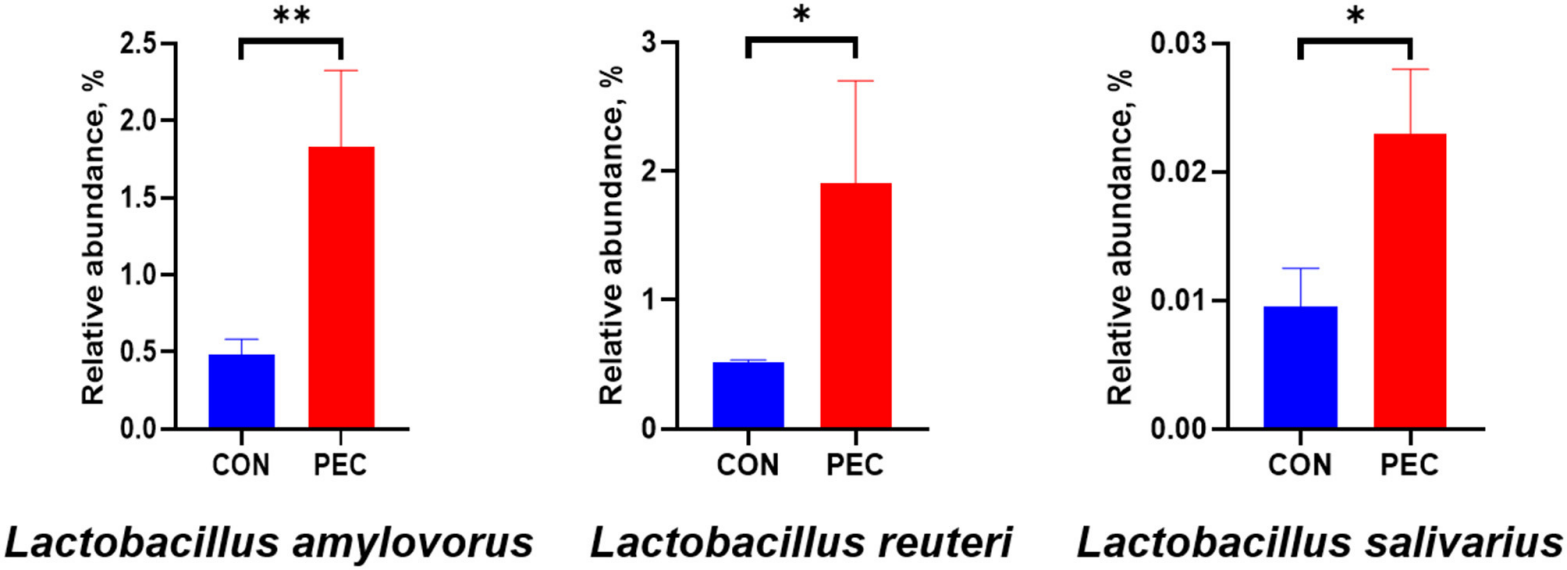
Relative abundance of sow vaginal *Lactobacillus*. CON, control group; PEC, pectin group. *n* = 9. ^*^P < 0.05, ^**^P < 0.01.

At the phylum level, Verrucomicrobiota was notably increased in PEC (*P* < 0.05, Supplementary Table 2), while at the genus level, the relative abundance of *Lactobacillus* (*P* < 0.05) and *UCG-005* (*P* < 0.05) in vagina microbiota were increased notably in PEC sows at day 110 of pregnancy (Table 2). At the species level, the relative abundance of *Lactobacillus amylovorus* was significantly higher (*P* < 0.01) in the PEC group than in the CON group. Meanwhile the abundance of *Lactobacillus reuteri* and *Lactobacillus salivarius* was also increased significantly (*P* < 0.05) in the PEC group than in the CON group (Figure 3).

**Table 2:** Relative abundance of top 20 bacterial genera of sow vaginal microbiota.

**Genus (%)**	**Treatment**		***P*-value**
	**Control**	**Pectin**	
*Clostridium_sensu_stricto _1*	7.50 ± 3.49	7.60 ± 3.78	1.000
*Staphylococcus*	5.84 ± 5.41	6.82 ± 3.75	0.609
*Bradyrhizobium*	6.17 ± 5.04	4.90 ± 3.87	0.798
*Lactobacillus*	1.85 ± 0.50	5.72 ± 4.44	0.015
*Terrisporobacter*	3.45 ± 1.07	3.17 ± 1.04	0.702
*Burkholderia-Caballeronia-Paraburkholderia*	3.73 ± 2.88	2.89 ± 2.46	0.609
*Streptococcus*	3.44 ± 5.96	2.54 ± 1.66	0.201
*Rhodococcus*	3.23 ± 2.80	1.99 ± 1.37	0.702
*Turicibacter*	2.95 ± 1.38	2.19 ± 1.14	0.443
*Psychrobacter*	2.47 ± 3.56	2.65 ± 5.93	0.798
*Ralstonia*	3.19 ± 2.44	1.78 ± 1.58	0.371
*norank_f__Muribaculaceae*	2.03 ± 3.68	2.86 ± 3.30	0.097
*Romboutsia*	2.28 ± 1.12	2.04 ± 1.10	1.000
*UCG-005*	1.29 ± 0.89	2.79 ± 1.19	0.021
*Christensenellaceae_R-7_group*	2.29 ± 1.91	1.17 ± 0.46	0.523
*Aerococcus*	1.67 ± 1.90	1.73 ± 0.81	0.609
*Bacteroides*	2.45 ± 4.09	0.93 ± 0.59	1.000
*Actinobacillus*	3.09 ± 5.84	0.27 ± 0.57	0.160
*Blautia*	1.34 ± 2.58	1.75 ± 2.78	0.074
*Clostridium_innocuum_group*	1.33 ± 3.12	1.46 ± 2.83	0.201

Data are expressed as means ± SEM, *n* = 9. Differences were considered significant at *P* < 0.05.

**Figure 4:**
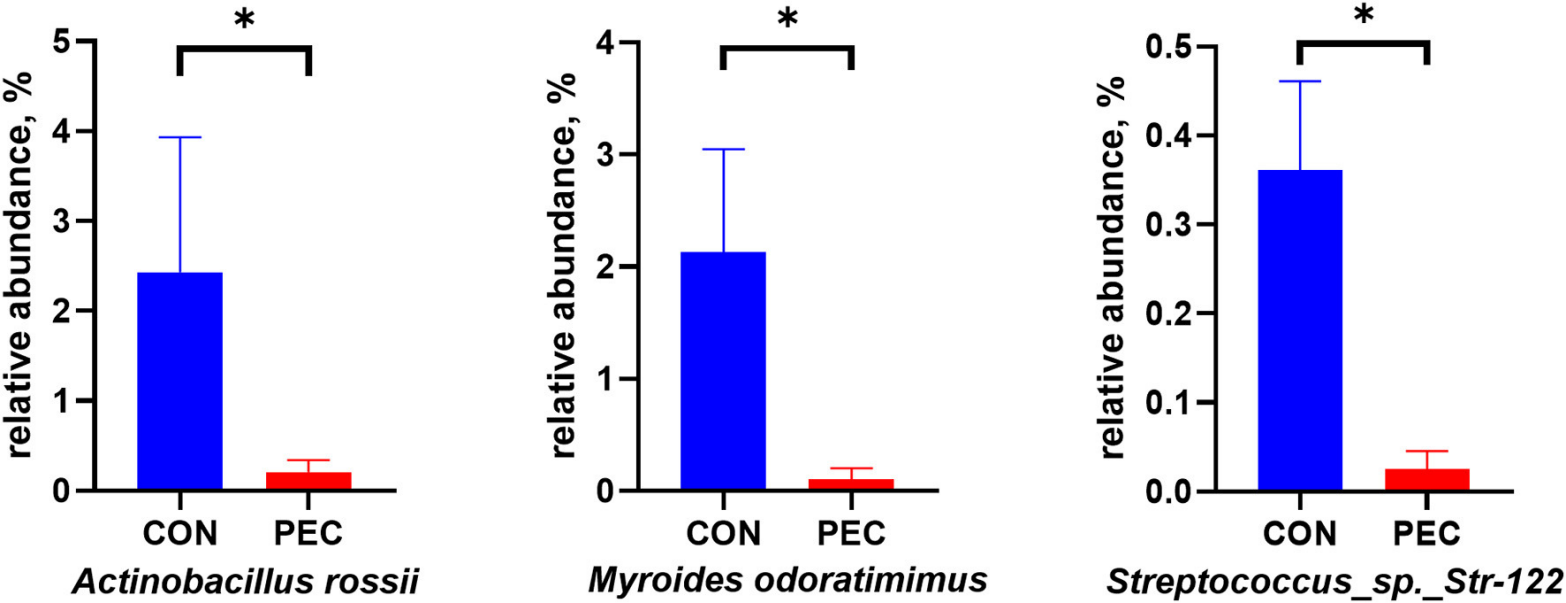
Relative abundance of pathogenic bacterium in sow vagina microbiota. CON, control group; PEC, pectin group. *n* = 9. ^*^P < 0.05.

The relative abundance of *Myroides odoratimimus, Actinobacillus rossii*, and *Streptococcus Str.122* was also significantly lower in the PEC group than those in the CON group (*P* < 0.05, Figure 4).

**Figure 5:**
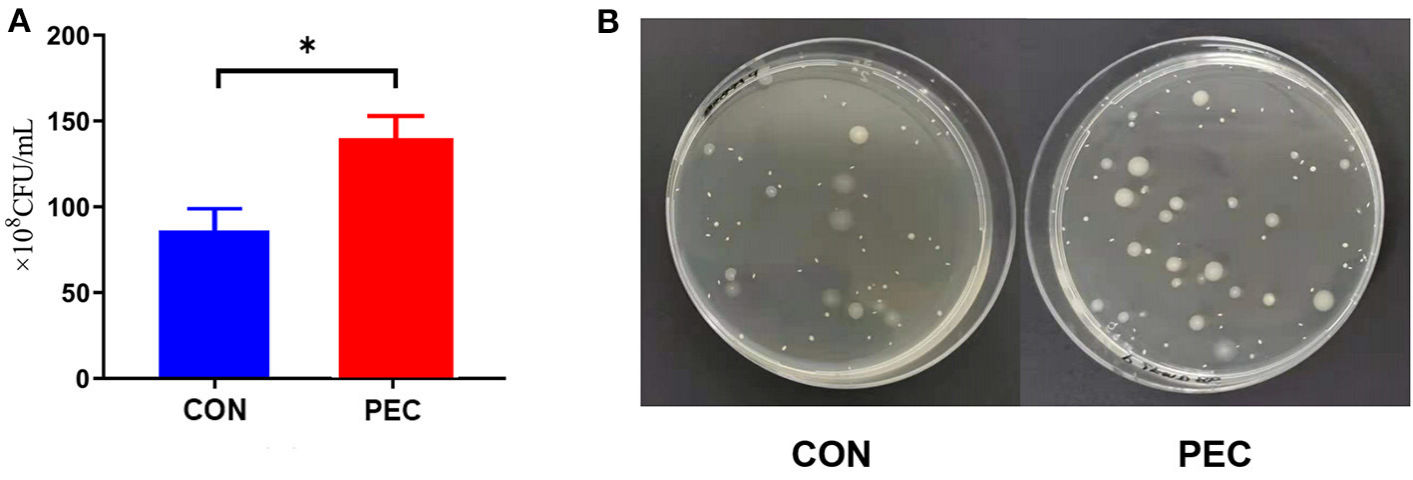
**(A)** Comparison of flat colony counting. **(B)** Pictures of the flats. CON, control group; PEC, pectin group; *n* = 9. ^*^P < 0.05.

Through flat colony culturing and counting with MRS agar, the concentration of vaginal *Lactobacillus* in the PEC group was detected significantly higher than in the CON group (*P* < 0.05, Figure 5).

### 3.4 Growth performance of suckling piglets

As shown in Table 3, no difference was determined in the growth performance of suckling piglets between treatments.

**Table 3:** Growth performance of suckling piglets.

**Item**	**Treatment**		**SEM**	***P*-value**
	**Control**	**Pectin**		
BW after cross-foster, kg	1.43	1.47	0.04	0.558
1st week BW, kg	2.52	2.43	0.05	0.416
1st week ADG, kg/d	0.16	0.14	0.01	0.164
2nd week BW, kg	3.55	3.51	0.11	0.855
1st^_^2nd week ADG, kg/d	0.15	0.16	0.01	0.678
BW at weaning, kg	5.17	5.03	0.14	0.614
2nd week weaning ADG, kg/d	0.23	0.22	0.01	0.454
ADG during suckling, kg/d	0.18	0.17	0.01	0.589

Data are expressed as means, *n* = 15. Differences were considered significant at *P* < 0.05.

### 3.5 Changes of colon microbiota of newborn piglets

**Figure 6:**
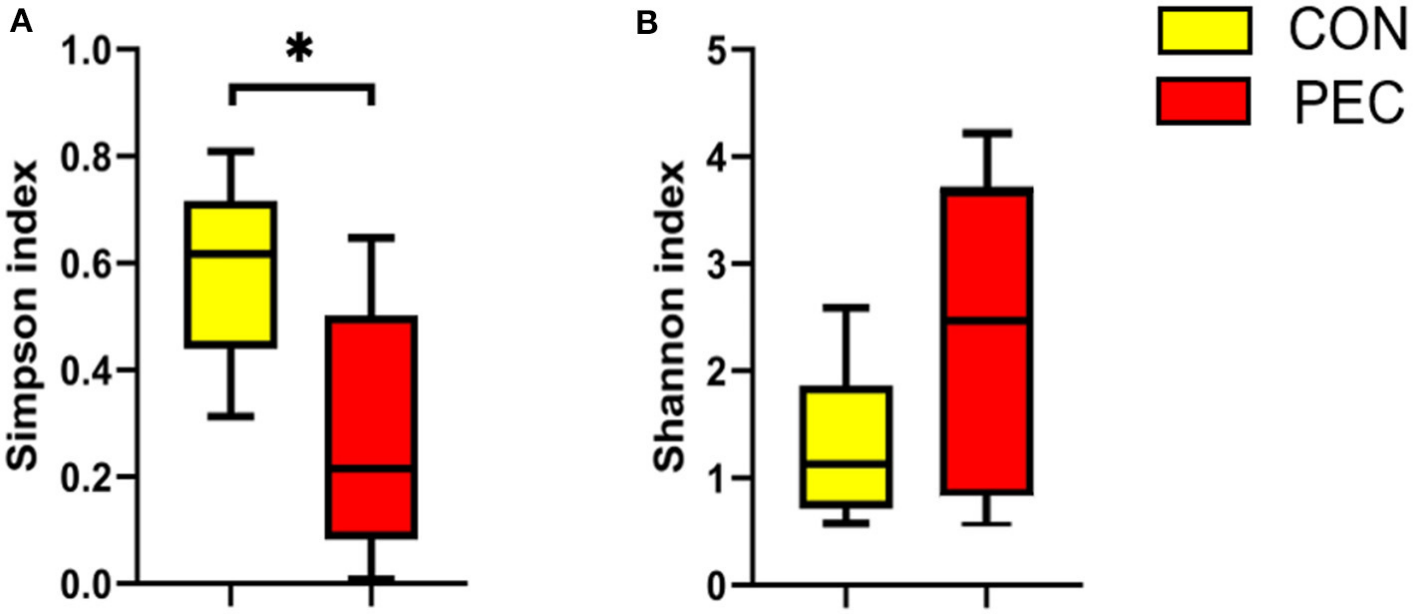
Effects of colon microbiota alpha-diversity of newborn piglets (*n* = 6). **(A)** Simpson index. **(B)** Shannon index. ^*^P < 0.05.

The α-diversity of colon microbiota of newborn piglets was significantly affected by maternal pectin supplementation. The Simpson index in the PEC group was notably lower than that in the CON group (*P* < 0.05, Figure 6A). Moreover, the Shannon index was higher in the PEC group than the CON group (*P* < 0.1, Figure 6B).

**Figure 7:**
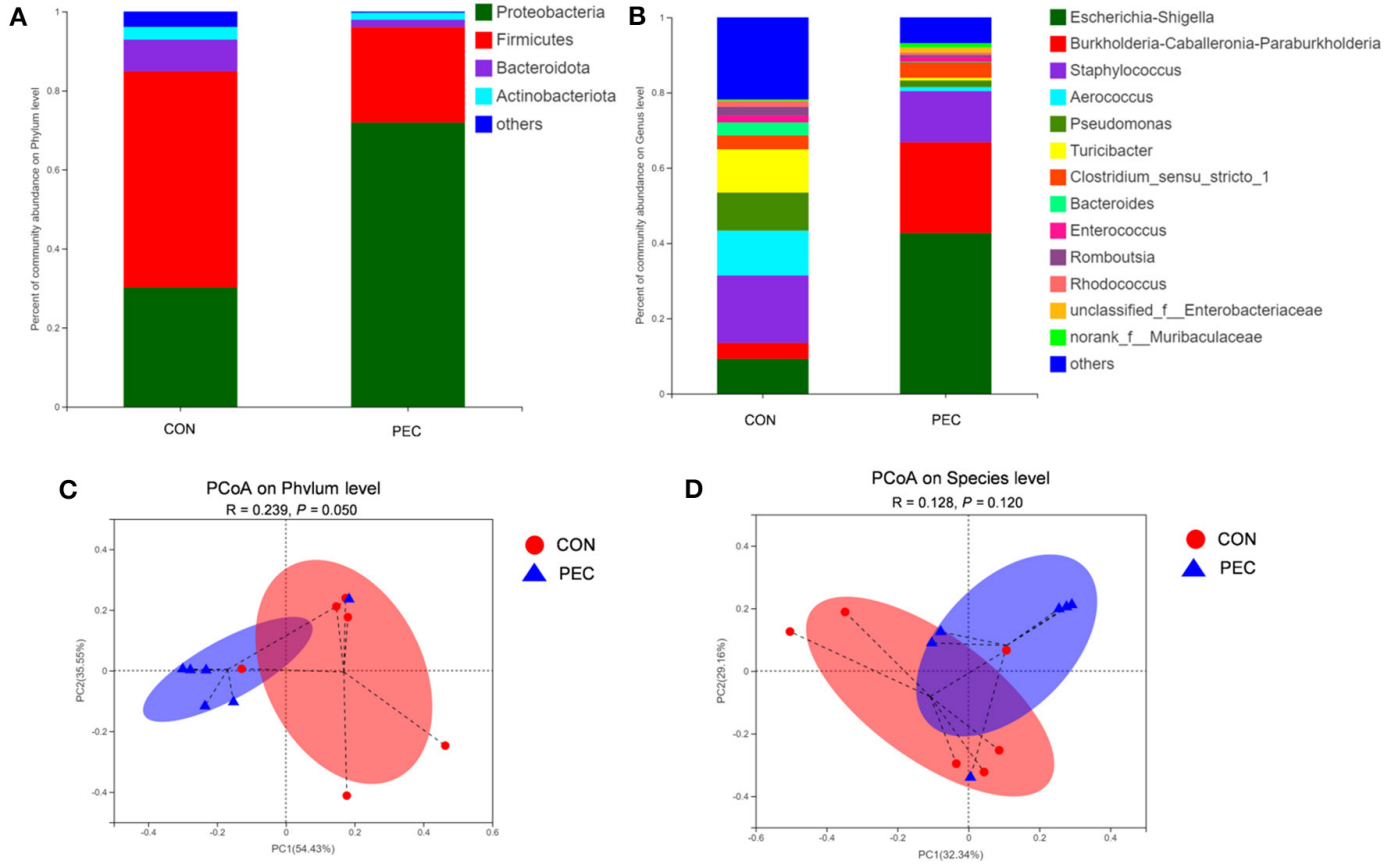
Comparison of the composition of genera in neonatal piglet colon content. Plot diagram of the relative abundance of microbiota on phylum level **(A)**, genus level **(B)**, phylum level **(C)**, and species level **(D)**. PCoA profile based on the weighted Unifrac distance of the OTUs in each sample. CON, control group; PEC, pectin group. *n* = 6.

The microbial composition in the colon of newborn piglets is obviously different at both the phylum (Figure 7A) and species (Figure 7B) level. Beta-diversity, evaluated by PCoA based on weighted Unifrac distance, revealed that the colon microbiota of newborn piglets formed a well-defined cluster and an obvious separation was seen at the phylum level (*P* = 0.050, Figure 7C). But the result at the species level displayed no differences (*P* > 0.1, Figure 7D).

At the phylum level, the relative abundance of Proteobacteria in piglets' colon was significantly lower in the PEC group, while the relative abundance of Firmicutes tended to be higher than in the CON group (Table 4).

**Table 4:** Relative abundance of main bacterial phyla in colon of newborn piglets.

**Phyla (%)**	**Treatment**		***P*-value**
	**Control**	**Pectin**	
Proteobacteria	71.36 ± 29.66	25.19 ± 22.15	0.045
Firmicutes	24.79 ± 30.14	53.45 ± 31.13	0.093
Bacteroidota	1.81 ± 2.74	9.66 ± 14.55	0.298
Actinobacteriota	1.77 ± 2.16	8.26 ± 14.1	0.689
Desulfobacterota	0.02 ± 0.05	0.69 ± 1.60	0.532
Chloroflexi	0.03 ± 0.04	0.62 ± 1.25	0.684
Acidobacteriota	0.02 ± 0.03	0.38 ± 0.77	0.532
Fusobacteriota	0.04 ± 0.07	0.25 ± 0.40	0.370
Cyanobacteria	0.07 ± 0.11	0.17 ± 0.23	0.561
Patescibacteria	0.03 ± 0.05	0.17 ± 0.29	0.733

Data are expressed as means ± SEM, *n* = 6. Differences were considered significant at *P* < 0.05.

### 3.6 Changes of plasma inflammatory cytokines of the newborn piglet

**Figure 8:**
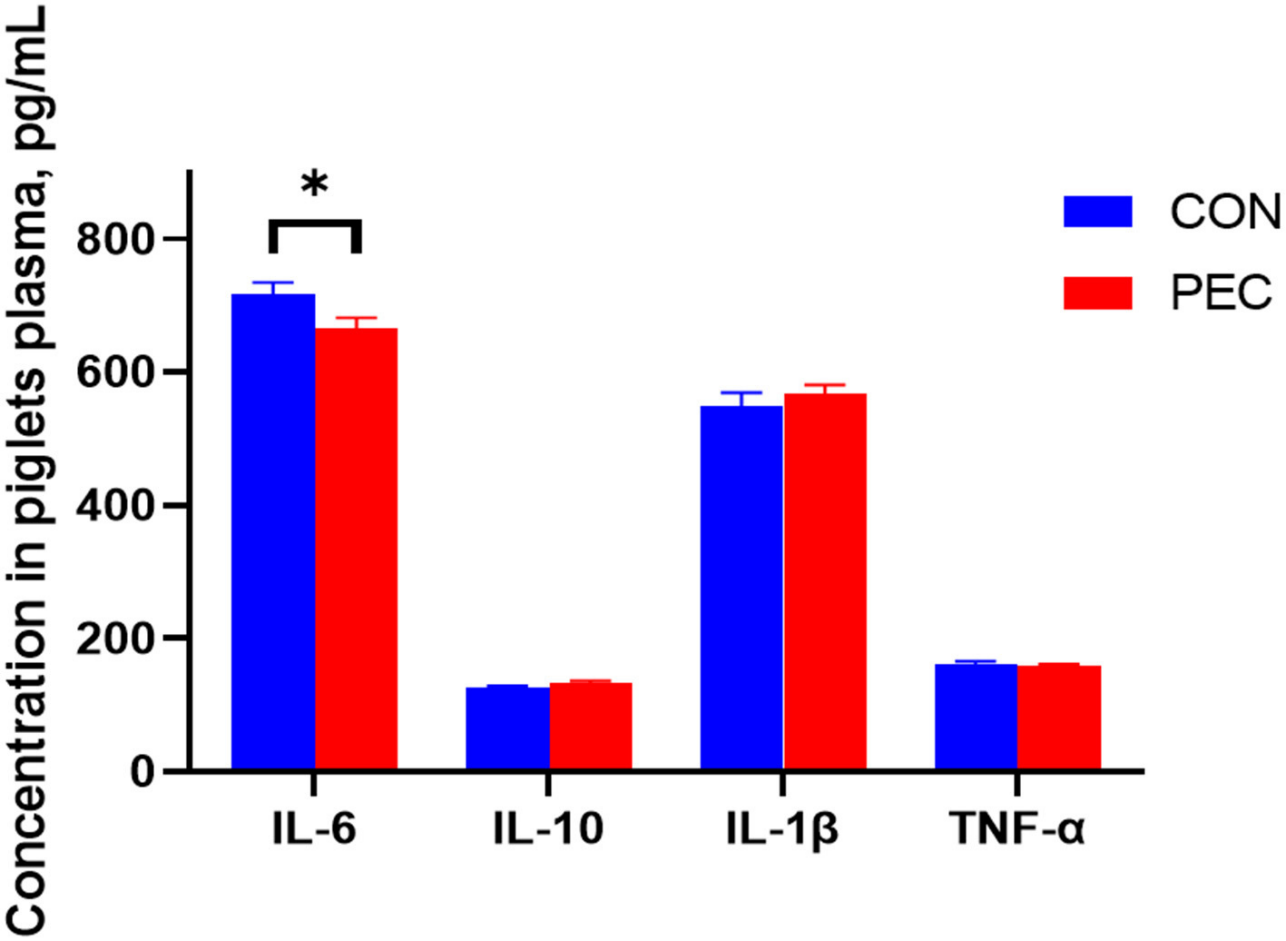
Effects of dietary pectin in pregnant sow diets on the plasma inflammatory cytokines of newborn piglets. IL-1β, Interleukin 1β; IL-6, Interleukin 6; IL-10, Interleukin 10; TNF-α, tumor necrosis factor alpha. Values are mean ± standard error. *n* = 6. ^*^P < 0.05.

The concentration of IL-6 in the plasma of newborn piglets was notably decreased in the PEC group than in the CON group (*P* < 0.05, Figure 8).

### 3.7 Changes of relative mRNA expression in newborn piglet intestines

**Figure 9:**
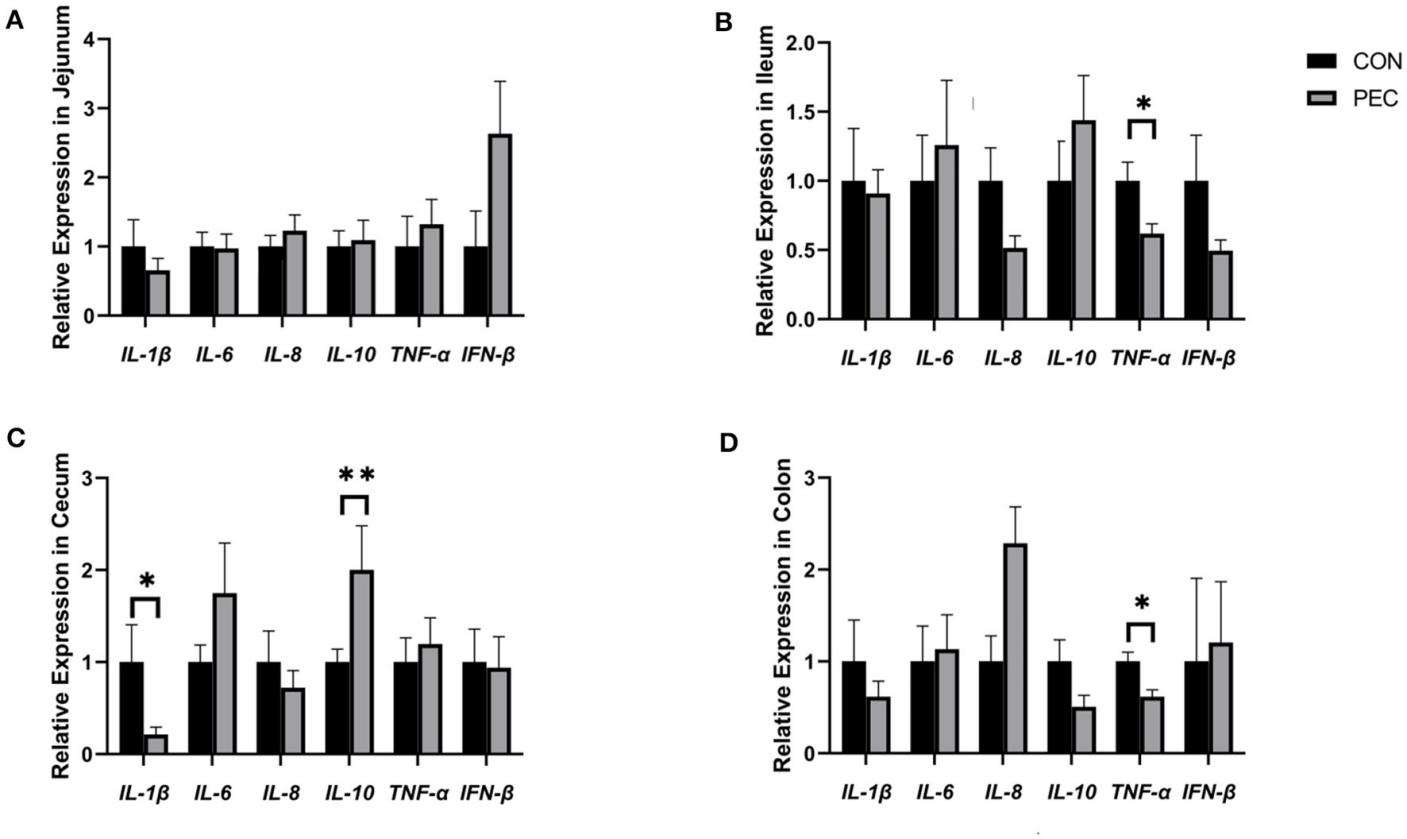
Effects of piglets' intestinal cytokinesis relative expression of mRNA: **(A)** jejunum; **(B)** ileum; **(C)** cecum; **(D)** colon. IL-1β, Interleukin 1β; IL-6, Interleukin 6; IL-8, Interleukin 8; IL-10, Interleukin 10; TNF-α, Tumor Necrosis Factor alpha; IFN-β, Interferon-beta; CON, newborn of control; PEC, newborn of pectin. Values are mean ± standard error. *n* = 6. ^*^P < 0.05, ^**^P < 0.01.

The effects of gestational dietary pectin supplementation on the relative mRNA expression of cytokine in the intestine of piglets are shown in Figure 9. In jejunum, no difference was detected (Figure 9A). But in ileum, the relative mRNA expression of TNF-α was found to be significantly higher in the CON group than in the PEC group (*P* < 0.05; Figure 9B). The relative IL-10 mRNA expression in the cecum was notably higher in the PEC group than in the CON group (*P* < 0.01). IL-1β was found to be expressed lower in the PEC group compared to the CON group (*P* < 0.05; Figure 9C). The relative expression of TNF-α in the colon (Figure 9D) was significantly lower (*P* < 0.05) in the PEC group than in the CON group, while in the CON group, the expression of IL-10 was higher (*P* < 0.1).

### 3.8 Changes in the intestinal development of newborn piglets

The results of morphometric measurements in the duodenum, jejunum, and ileum in neonatal piglets are shown in Table 5. The villus height and V/C ratio of duodenum, ileum, and jejunum in the PEC group was extremely significantly higher than that in the CON group (*P* < 0.001). The crypt depth of ileum in the CON group was higher than in the PEC group (*P* < 0.1). Meanwhile, the muscular layer thickness of duodenum in the PEC group was markedly higher than that in the CON group (*P* < 0.05).

**Table 5:** Effects of the morphometric measurements in the duodenum, jejunum, and ileum in newborn piglets.

**Item**	**Treatment**		**SEM**	***P*-value**
	**Control**	**Pectin**		
**Duodenum**				
Villus height, μm	408.92	490.52	6.22	< 0.001
Crypt depth, μm	122.92	122.52	1.39	0.889
Villus/crypt ratio	3.45	4.20	0.07	< 0.001
Muscular layer thickness, μm	151.41	159.20	1.69	0.023
**Ileum**				
Villus height, μm	603.77	671.30	7.67	< 0.001
Crypt depth, μm	88.51	84.16	1.10	0.054
Villus/crypt ratio	7.12	8.44	0.13	< 0.001
Muscular layer thickness, μm	143.65	145.73	2.01	0.615
**Jejunum**				
Villus height, μm	632.72	704.00	10.06	< 0.001
Crypt depth, μm	70.68	68.74	0.97	0.322
Villus/crypt ratio	9.34	10.59	0.18	0.001
Muscular layer thickness, μm	86.43	87.43	1.31	0.731

Data are expressed as means, *n* = 6. Differences were considered significant at *P* < 0.05.

## 4 Discussion

### 4.1 β-G and E2 influenced by dietary pectin supplementation

The estrobolome has been defined as the gene repertoire of the microbiota in the gut that are capable of regulating estrogens. The conjugated estrogens existing in the gut can be deconjugated by bacterial secreted β-G into estrogen, which will be reabsorbed through the epithelial cells to the blood stream (24, 25), and distributed to different distant organs like the epithelium in the vagina (26). Microorganisms like *Faecalibacterium prausnitzii, Ruminococcus, Clostridium*, and so on demonstrated their ability to produce β-G (27–30). The observed increase in fecal β-G activity within the pectin group corroborates our hypothesis. This finding aligns with Licht et al. (19), who demonstrated that incorporating 7% apple pectin into rat's diet for 14 weeks substantially elevates β-G activity in rat feces. Similarly, other researchers (18) contended that in the presence of a pectin–protein complex isolated from cabbage, the activity of β-G was significantly enhanced *in vitro*. Furthermore, the addition of wheat flour aleurone, a fermentable dietary fiber sourced from the outer layer of wheat grains, has been reported to significantly boost β-G activity in the cecum of rats (12).

In our study, we observed a significant increase in the concentration of estradiol (E2) in plasma by day 110 of gestation with the supplementation of pectin to sows during gestation. The elevation in the E2 levels may be influenced by the activity of β-G, which was modified by alterations in the gut microbiota as mentioned. This is in line with the findings in postmenopausal women, who, despite their ovaries not producing estrogen, exhibit higher E2 concentrations in their blood when their diets are rich in soluble fiber (31). Conversely, some studies have demonstrated that increasing composition of fiber in diets can reduce the level of free E2 in the bloodstream (32–34). This theory is plausible as fiber intake can increase stool bulk, facilitating the premature excretion of conjugated estrogen metabolites that originate from enterohepatic circulation. For example, Jiang (35) reported that crude fiber intake can remarkably increase the concentration of free E2 in feces, but not in saliva, which indicates that fiber intake might facilitate the deconjugation of E2 in the hindgut, but estrogen was excreted in the expanded feces as a result of insoluble fiber before being reabsorbed into the bloodstream.

### 4.2 Ingestion of pectin increased *Lactobacillus* in the vagina

During pregnancy, E2 can not only maintain the pregnancy status but also improve the vaginal microbiota to be dominated by *Lactobacillus*. Elevated levels of E2 promote the maturation and deposition of glycogen in vaginal epithelial cells. Glycogen from exfoliated and lysed epithelial cells is catabolized by α-amylase in the vaginal lumen to smaller polymers that are subsequently metabolized to lactic acid by *Lactobacillus*. Lactic acid acidifies the vaginal milieu, favoring the proliferation of *Lactobacillus* and inhibiting the growth of infection-associated organisms (36–40). Thus, some studies aimed to improve vaginal microbes during pregnancy through nutrition methods. Yang (41) tried to improve the vaginal microbiota of pregnant women by giving them *Lactobacillus rhamnosus GR-1*, and *Lactobacillus reuteri RC-14* orally, but they were found to be ineffective in increasing microbes. However, supplying *Lactobacillus rhamnosus GR-1* and *Lactobacillus reuteri RC-14* in capsules to pregnant women was suggested to significantly reduce vaginal *Streptococcus agalactiae* colonization (42). But almost all of the above studies were only providing pregnant women with probiotics directly and did not look into the effects of non-microbial prebiotics on vaginal microbiota, while the present study indicates that it is possible to increase the relative abundance of *Lactobacillus* in the vagina of sows with pectin-supplied diet.

Two species of bacteria were also found to be reduced remarkably in vagina in the PEC group. *Myroides odoratimimus* is associated with urinary tract inflammation in humans (43, 44), which indicated that supplying sows with pectin during gestation may decrease the risk of urinary tract inflammation. Mayor et al. (45) certified that *Actinobacillus rossii* was isolated from aborted piglets, uterus, and vaginal discharge since 1989 in USA. Holyoake and Thompson (46) isolated *A. rossii* from an aborted piglet as well, which means these abortions are probably caused by *A. rossii*, or there might be some relationship between abortion and *A. rossii*. Therefore, we further explored the intestinal health of the new piglet generation.

### 4.3 Pectin supplementation during pregnancy improves the gut microbiota of the offspring

Providing pectin-containing diet to the sows during pregnancy has a significant impact on the colonic microbial diversity and their composition in the neonatal piglets. Compared with the CON group, the colonic flora of piglets in PEC has a higher Shannon index and a lower Simpson index, which means a higher microbial diversity. The β-diversity of the colonic microbiota of piglets from each group was found with a significant discrimination through PCoA. The relative abundance of phylum Proteobacteria and species *Escherichia–Shigella*, both generally regarded as harmful bacteria, was lower in the PEC group than in the CON group. In addition, studies have shown that part of mother's vaginal microbiota will be colonized in the intestines of the offspring through vaginal delivery (47). And in the sows and piglets, the mode of delivery and early nutrition modulate microbial colonization (48). This suggests that if the mother has vaginal microbiota dominated by probiotics before giving birth, the microbiota of newborn intestine may be colonized with probiotics and may provide a better condition to the offspring. Surprisingly, *Streptococcus_suis* enriched in the CON sows' vagina was found to be notably higher in the colon of the CON offspring, but *Lactobacillus*, which also were notably enriched in the vagina of sows in the PEC group did not show any difference in the colon of piglets from both groups. This may indicate that the transmission of microorganisms between sows and piglets does not entirely depend on the contact with vaginal mucus during vaginal delivery. Recently, sequencing has indicated that placenta and umbilical cord blood is not sterile, and the microbiota within the neonate's meconium shares significant similarity with that of the placenta, thus suggesting that maternal transfer of microbiota is possible and might occur during gestation (49–51). Therefore, the maternal microbiota plays a vital role in the composition of the intestinal flora of the offspring.

### 4.4 Pectin supplementation improves the development and anti-inflammatory ability of newborn piglets

Maternal nutrition is known to impact the development of the intestine (52), with the morphology of the intestine serving as a key indicator of its health. Villus height, in particular, plays a crucial role in determining the nutrient absorption capacity of the small intestine. The ratio of villus height to crypt depth (V/C) is regarded as a reliable measure for assessing the nutrient absorption potential of the small intestine. In this study, offspring of mothers supplemented with pectin during pregnancy exhibited increased villus height and V/C ratio, suggesting an enhanced ability to absorb nutrients. Similarly, existing research indicates that maternal diets high in soluble fiber during gestation can alter the intestinal microbiota, enhance growth performance, and reduce intestinal permeability in piglets (53).

Accumulating evidence indicates that maternal inflammation also has long-term consequences for offspring by affecting the intrauterine environment (54). In the present study, we found that piglets from the PEC group exhibited lower plasma IL-6 levels. IL-6 is a pleiotropic cytokine participating in the physiology of virtually every organ system (55). We can not only find the change of inflammatory cytokines in the piglet plasma but also in their intestine. Although no differences were found in the jejunum, they gradually emerged from the ileum. The improvement of intestinal immune function of newborn piglets is thought to be mediated by microorganisms (56, 57). A study showed a similar result of a maternal soluble fiber diet increasing the plasma concentrations of anti-inflammatory factors, IL-10, in the offspring, and both sows and piglets were found to have lower plasma TNF-α levels with soluble fiber intake (21).

## 5 Conclusion

Firstly, dietary pectin supplementation can be of benefit to sow vaginal microbiota and increase the relative abundance of *Lactobacillus*. Secondly, pectin supplementation for sow during gestation can improve the development and the anti-inflammatory ability of the intestine of newborn piglets.

## Data availability statement

All obtained raw sequence datasets have been uploaded to the NCBI Sequence Read Archive (SRA) with the accession number: PRJNA754492.

## Ethics statement

The animal study was approved by Animal Care and Use Committee of Sichuan Agricultural University. The study was conducted in accordance with the local legislation and institutional requirements.

## Author contributions

JH: Writing – original draft, Writing – review & editing. JZ: Writing – review & editing. YH: Writing – review & editing. SL: Writing – review & editing. LH: Writing – review & editing. XJ: Writing – review & editing. LC: Writing – review & editing. ZF: Writing – review & editing. BF: Writing – review & editing. YL: Writing – review & editing. SX: Writing – review & editing. JL: Writing – review & editing. DW: Writing – review & editing, Funding acquisition, Writing – original draft.
