# Supplementary material for: Benefits of maternal pectin supplementation in gestation diet on vaginal microbiota of sows and intestinal health of newborn piglets
Source: Front Vet Sci. 2024 Jun 4;11:1392399. doi: 10.3389/fvets.2024.1392399 (PMC11183816; doi:10.3389/fvets.2024.1392399)
Supplement: Supplementary file 1 [file Table_1.DOCX]

**Table S1**. Primer sequences of the target and reference genes

| Genes symbol | Nucleotide sequence of primers (5'–3') | Accession |
| --- | --- | --- |
| *β-actin* | F: GGCGCCCAGCACGAT  R: CCGATCCACACGGAGTACTTG | XM_021086047.1 |
| *IL-1β* | F: TCTGCCCTGTACCCCAACTG  R: CCAGGAAGACGGGCTTTTG | NM_214055.1 |
| *IL-6* | F: ATGCTTCCAATCTGGGTTCAA  R: CACAAGACCGGTGGTGATTCT | NM_001252429.1 |
| *IL-8* | F: GCAAGAGTAAGTGCAGAACTTCGA  R: GGGTGGAAAGGTGTGGAATG | NM_213867.1 |
| *IL-10* | F: CAGATGGGCGACTTGTTGCT  R: GGCAACCCAGGTAACCCTTAA | NM_214041.1 |
| *TNF-α* | F: CGACTCAGTGCCGAGATCAA  R: CCTGCCCAGATTCAGCAAAG | NM_214022.1 |

^1^F = forward primer sequence (5' → 3'), and R = reverse primer sequence (5' → 3').

^2^Gene: *IL-1β*=Interleukin 1β; *IL-6*=Interleukin 6; *IL-8*=Interleukin 8; *IL-10*=Interleukin 10; *TNF-α*=Tumour Necrosis Factor alpha; *IFN-β*= Interferon-beta
