# Supplementary material for: Benefits of maternal pectin supplementation in gestation diet on vaginal microbiota of sows and intestinal health of newborn piglets
Source: Front Vet Sci. 2024 Jun 4;11:1392399. doi: 10.3389/fvets.2024.1392399 (PMC11183816; doi:10.3389/fvets.2024.1392399)
Supplement: Supplementary file 2 [file Table_2.DOCX]

**Table S2**. Relative abundance of main bacterial phyla of sow vaginal microbiota

| Phyla (%) | Groups | | *P*-value |
| --- | --- | --- | --- |
|  | Control | Pectin |  |
| Firmicutes | 50.800 | 58.740 | 0.201 |
| Proteobacteria | 26.630 | 21.170 | 0.307 |
| Bacteroidota | 8.903 | 8.752 | 0.443 |
| Actinobacteriota | 8.831 | 7.961 | 0.443 |
| Spirochaetota | 1.240 | 1.041 | 0.898 |
| Cyanobacteria | 1.081 | 0.745 | 0.371 |
| Campilobacterota | 0.967 | 0.614 | 0.055 |
| Fusobacteriota | 0.775 | 0.112 | 0.250 |
| Verrucomicrobiota | 0.115 | 0.274 | 0.011* |
| Patescibacteria | 0.095 | 0.244 | 0.160 |

*Denotes *P* < 0.05 and **denotes *P* < 0.01.
